# Special Issue: “Pharmacological Strategies and Molecular Mechanisms Associated with the Novel Nephroprotective Treatments”

**DOI:** 10.3390/ijms26199556

**Published:** 2025-09-30

**Authors:** Michele Provenzano

**Affiliations:** 1 Department of Pharmacy, Health and Nutritional Sciences, University of Calabria, 87036 Rende, Italy; michele.provenzano@unical.it; 2 Nephrology, Dialysis and Transplant Unit, “SS. Annunziata” Hospital, 87100 Cosenza, Italy

In recent years, research efforts have resulted in a significant increase in the number of therapies available for kidney disease care [1]. These efforts have been prompted by the rising prevalence of acute and chronic kidney disease (CKD) worldwide [2]. Chronic kidney disease refers to the condition of kidney damage lasting at least three months. There are a range of possible initial causes, including immunological, genetic, hemodynamic, infection-related, and metabolic factors. Regardless of the cause, the onset of CKD leads to an increased risk of negative events during the patient’s lifetime, such as fatal or non-fatal cardiovascular (CV) events, death from any cause, or kidney failure, i.e., the final stage of CKD, which is often not responsive to medical therapies and requires renal replacement treatment. Furthermore, CKD is a complex disease; multiple damage mechanisms are active at the same time and play key roles in establishing the patient’s prognosis [3,4,5,6]. For this reason, the molecular targets for novel drugs encompass different types of cells and tissues. One of the most frequently investigated targets is the modulation or inhibition of the renin–angiotensin–aldosterone system (RAAS). The RAAS is a “ancient” network of biological factors that guided the evolution of mammalians by allowing us to switch from a water to a land environment. It involves renin produced by the kidneys, angiotensin I and II, which are activated by angiotensin-converting enzyme (ACE), and aldosterone secreted from the zona glomerulosa of the adrenal cortex. The RAAS is involved in regulating blood pressure in humans and defending against potentially life-threatening water and sodium loss. It has been shown that the hyperactivation of the RAAS promotes damage in vital organs such as the kidneys, blood vessels, and heart, and this is mainly mediated by chronic fibrosis [7,8,9,10,11]. Ksiazek and colleagues discuss pharmacological strategies to block the RAAS and improve prognosis in patients with CKD [12]. In particular, they focus on the older ACE inhibitors and angiotensin receptor blockers, which are currently the most commonly used drugs in patients with CKD, and then move onto non-steroidal mineralocorticoids receptor antagonists (nsMRA), inhibitors of aldosterone synthase (ASI), an Aminopeptidase A inhibitor (APAi) and angiotensinogen suppressor. These drugs intervene in various steps in the RAAS sequence and can modulate the suppression of the system, improve blood pressure control, and reduce kidney function decline [13,14,15,16] (Figure 1).

Research on the prevention and treatment of kidney fibrosis is *gaining momentum*. In this Special Issue, Hou et al. report on the role of Indoxyl sulfate (IS) in kidney fibrosis [17]. They simulated fibrosis conditions via unilateral ureteral obstruction in mice and assessed fibrosis after 14 days. Interestingly, they found that the degree of fibrosis reduced in mice with a lower accumulation of IS, opening up novel avenues for future research on this topic. The intensification of the use of RAAS inhibitors underscores the importance of reducing blood pressure in CKD patients: blood pressure (especially systolic blood pressure) is a significant predictor of kidney function decline and CV events [18,19,20]. Moreover, CKD patients are often not completely responsive (i.e., their blood pressure does not reach the target range) with only one drug. A potent blood pressure-lowering drug class comprises calcium channel blockers (CCBs). These drugs are largely used in CKD patients due to their efficacy [21,22]. In this Special Issue, Hajdys and Colleagues discuss several pharmacological aspects of lercanidipine, an L- and T-type CCB [23]. Lercanidipine can reversibly block L-type CC expression in all excitable cells and exert antihypertensive and anti-ischemic effects. Moreover, it shows a nephroprotective effect through the concomitant blockage of L- and T-type channels in the kidney arterioles. It is a promising drug for CKD patients, and it also exerts general antioxidative and antiatherosclerosis effects that warrant further attention.

The final two articles in this Special Issue discuss aspects of acute kidney injury (AKI). In their paper, Chen et al. summarize the interesting association between non-alcoholic fatty liver disease (NAFLD) and AKI [24,25]. A number of dysregulating factors such as steatosis, insulin resistance, inflammation, and hepatic factors can alter the cross-talk between vital organs and can, in part, explain the onset of AKI in NAFLD patients [26,27,28,29]. This is a timely paper, since the role of metabolism in kidney disease, along with lifestyle and dietary interventions, is of increasing interest. New ideas around metabolism are also replacing past beliefs, and thus investigations in this area represent a research priority. On the other hand, Torso and colleagues explored the problem of AKI in cisplatin therapies in head and neck cancers [30]. They particularly examined the role of kidney injury molecule-1 (KIM-1) in predicting AKI after cisplatin according to the common classifications of AKI, namely, the Common Terminology Criteria for Adverse Events (CTCAE), Risk, Injury, Failure, Loss, and End-stage kidney disease (RIFLE), and Acute Kidney Injury Network (AKIN). Interestingly, they found an association between KIM-1 levels and the CTCAE and AKIN classification of AKI, opening up possibilities for further research around these biomarkers, which is especially relevant right now, as there is significant excitement around the development of biomarkers and their implementation in clinical practice [31,32].

All the contributions to this Special Issue show the challenges encountered in nephrology and introduce questions that must be answered in order for clinical and research aims to be achieved.

## Figures and Tables

**Figure 1:**
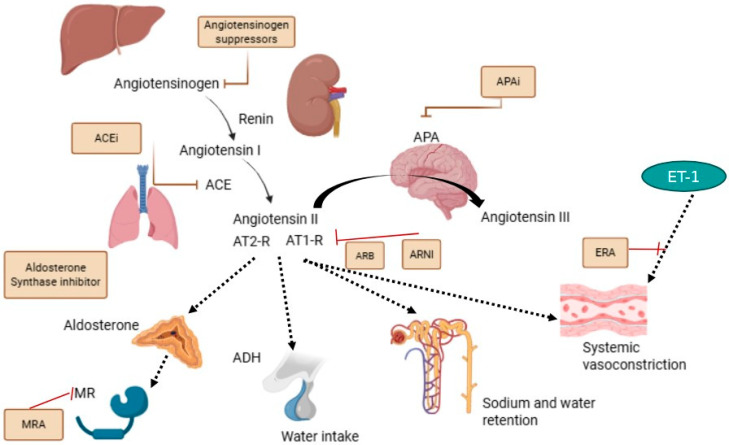
This figure illustrates novel pharmacological agents targeting the renin–angiotensin–aldosterone system and their mechanisms of action. Angiotensin II type 1-receptor blockers (ARBs) inhibit angiotensin II from binding to its type 1 receptor (AT1-R), thus counteracting its primary effects. Angiotensin II type 1-receptor–neprilysin inhibitors (ARNIs) combine the action of an ARB, i.e., blocking AT1-R, with the inhibition of neprilysin. Aminopeptidase A inhibitors (APAis) prevent aminopeptidase A (APA) from converting angiotensin II into angiotensin III. Mineralocorticoid-receptor antagonists (MRAs) block the mineralocorticoid receptor (MR), which is where aldosterone normally binds to induce increased sodium and water reabsorption. Additionally, Endothelin-receptor antagonists (ERAs) act by blocking the binding of the vasoconstrictive Endothelin-1 (ET-1) to Endothelin-receptors A and/or B on smooth muscle cells. The pathway targeted by this mechanism is related to, but distinct from, direct RAAS cascade.
